# Melatonin Ameliorates Autophagy Impairment in a Metabolic Syndrome Model

**DOI:** 10.3390/antiox10050796

**Published:** 2021-05-18

**Authors:** Adrián Santos-Ledo, Beatriz de Luxán-Delgado, Beatriz Caballero, Yaiza Potes, Susana Rodríguez-González, José Antonio Boga, Ana Coto-Montes, Marina García-Macia

**Affiliations:** 1 Institute of Neurosciences of Castilla y León-INCYL, Institute of Biomedical Research of Salamanca-IBSAL, Cell Biology and Pathology, University of Salamanca, 37007 Salamanca, Spain; santosledo@usal.es; 2 Centre for Tumour Biology, Barts Cancer Institute-Queen Mary, University of London, John Vane Science Centre, Charterhouse Square, London EC1M 6BQ, UK; b.luxandelgado@gmail.com; 3 Departamento de Morfología y Biología Celular, Área de Biología Celular, Facultad de Medicina, Universidad de Oviedo, Julián Clavería s/n, 33006 Oviedo, Spain; caballerobeatriz@uniovi.es (B.C.); potesyaiza@uniovi.es (Y.P.); gusatan69@gmail.com (S.R.-G.); 4 Instituto de Investigación Sanitaria del Principado de Asturias (ISPA), Av. del Hospital Universitario, s/n, 33011 Oviedo, Spain; 5 Servicio de Microbiología, Hospital Universitario Central de Asturias, Celestino Villamil s/n, 33006 Oviedo, Spain; joseantonio.boga@sespa.es; 6 Institute of Biomedical Research of Salamanca, University Hospital of Salamanca, 37007 Salamanca, Spain; 7 Institute of Functional Biology and Genomics, University of Salamanca, CSIC, 37007 Salamanca, Spain; 8 Centro de Investigación Biomédica en Red Sobre Fragilidad y Envejecimiento Saludable (CIBERFES), Instituto de Salud Carlos III, 28029 Madrid, Spain

**Keywords:** melatonin, metabolic syndrome, autophagy, lipophagy, Harderian gland

## Abstract

Metabolic syndrome is a global health problem in adults and its prevalence among children and adolescents is rising. It is strongly linked to a lifestyle with high-caloric food, which causes obesity and lipid metabolism anomalies. Molecular damage due to excessive oxidative stress plays a major role during the development of metabolic syndrome complications. Among the different hormones, melatonin presents strong antioxidant properties, and it is used to treat metabolic diseases. However, there is not a consensus about its use as a metabolic syndrome treatment. The aim of this study was to identify melatonin effects in a metabolic syndrome model. Golden hamsters were fed with 60% fructose-enriched food to induce metabolic syndrome and were compared to hamsters fed with regular chow diet. Both groups were also treated with melatonin. Fructose-fed hamsters showed altered blood lipid levels (increased cholesterol and LDL) and phenotypes restored with the melatonin treatment. The Harderian gland (HG), which is an ideal model to study autophagy modulation through oxidative stress, was the organ that was most affected by a fructose diet. Redox balance was altered in fructose-fed HG, inducing autophagic activation. However, since LC3-II was not increased, the impairment must be in the last steps of autophagy. Lipophagy HG markers were also disturbed, contributing to the dyslipidemia. Melatonin treatment improved possible oxidative homeostasis through autophagic induction. All these results point to melatonin as a possible treatment of the metabolic syndrome.

## 1. Introduction

Current changes in lifestyle and eating behavior are increasing the prevalence of overweight and obesity, reaching the status of pandemic. These changes include sedentariness and an elevated consumption of high-calorie food and sugary beverages. Although obesity is a known risk factor for metabolic defects, associated problems like insulin resistance and diabetes also affect normal weight people. Metabolic syndrome (MetS), cluster abnormalities including abdominal obesity, insulin resistance, and dyslipidemia, increased blood pressure, pro-inflammatory states, and risk of cerebrovascular accidents [1]. MetS is also characterized by high levels of oxidative stress, which might play a fundamental role in its progression [2], since the accumulation of free radicals is a harmful process that can damage several cellular structures. This state, together with reduced antioxidant defenses [3], suggests that an oxidative imbalance might be very relevant to MetS.

The pineal melatonin is the capital endogenous synchronizer of the circadian rhythms in many organisms [4]. Likewise, melatonin as well as its metabolites, are well-known endogenous antioxidants [5] and they exert anti-inflammatory, antihyperlipidemic, and anti-hypertensive actions and modulates insulin secretion and action [6], mainly fostering the antioxidant system. Melatonin treatment ameliorates metabolic changes associated with obesity in rats fed a high-fat diet [7] or metabolic changes and hypertension associated with obesity in young Zucker diabetic fatty rats [1]. Melatonin was even suggested as a treatment for viral infections like Sars-Cov2 [8]. However, it is still unclear whether melatonin could ameliorate the pathological phenotype during MetS, induced by high-fructose intake in hamsters, which might better emulate the harmful effects of prepared foods and carbonated beverages [9].

The Syrian hamster Harderian gland (HG) is a tubule-alveolar orbital gland secreting lipid that lubricate the cornea [10]. This small gland presents many more functions including the production of pheromones [11], the participation in a pineal–gonadal axis [12], the synthesis of indolamines, as melatonin [13], and an important porphyrin production (which might regulate melatonin production [14]) that is stronger than that in the liver [15]. Moreover, due to the localization of the HG, porphyrins exposed to light produce reactive oxygen species (ROS) through photo-oxidation. Therefore, the gland is an excellent model to comprehend physiological oxidative stress and its control [16]. In previous reports we showed that damage caused by oxidative stress forces the gland to trigger autophagic processes to maintain vital functions, adapting to environmental stress [17]. We wondered if a similar scenario could occur after inducing MetS, and whether melatonin might present protective functions. 

Our aim was to identify melatonin effects in a metabolic syndrome model. Particularly, the melatonin role in the autophagic response to oxidative stress. Here, we showed that melatonin ameliorates the fructose-diet induced dyslipidemia. Furthermore, melatonin treatment partially restored impaired autophagy in the most affected organ through a fructose diet.

## 2. Material and Methods

### 2.1. Animals

Eight-week-old male Syrian hamsters (*Mesocricetus auratus*) (Harlan Interfauna Ibérica, Barcelona, Spain) were housed 2 per cage, during long days, with a 14:10 light:dark cycle (lights on daily from 07:00 to 21:00) at 22 ± 2 °C (*n* = 6 per experimental condition). Animals received water and a standard pellet diet, ad libitum. The Oviedo University Local Animal Care and Use Committee approved the experimental protocols. All experiments were carried out according to the Spanish Government Guide and the European Community Guide for Animal Care (Council Directive 86/609/EEC). After 1 month in the animal house, the hamsters were fed either a high-fructose diet (*n* = 12) (TD.89247, Harlan Interfauna Ibérica, Barcelona, Spain) or a regular chow diet (*n* = 12) for 4 weeks, following the Kasim-Karakas’ protocol to induce Metabolic Syndrome (MetS) [18]. Then, six hamsters of each diet were treated daily with 25 µg of melatonin for 15 days. Melatonin was dissolved in ethanol (final ethanol concentration, 0.5%) and injected subcutaneously (SQ) at Zeitgeber time (ZT) 10 (ZT 0 = onset of light). The controls received the same volume of saline (0.5% final ethanol concentration) under similar conditions, six animals for fifteen days. Weight and food intake was monitored weekly. Blood extraction was performed 1 day before harvest and the blood parameters (glucose, triglycerides, total cholesterol, HDL cholesterol and LDL cholesterol) were analyzed by routine laboratory tests at the Laboratory of Veterinary Analysis of Dr. Barba (Madrid, Spain). Hamsters were sacrificed and the Harderian glands were immediately removed, weighted, frozen in liquid nitrogen, and stored at −80 °C, until the experiments were performed. The other organs (brain, liver, muscle, heart, and white adipose tissue) were removed, weighted, and stored at −80 °C.

### 2.2. Isolation of Proteins

HGs (0.1 g) were homogenized using a Polytron homogenizer at 4 °C in 1 mL of lysis buffer (50 mM Tris/HCl, 150 mM NaCl at pH 7.4). The tissue homogenates were then centrifuged for 6 min at 3000 rpm at 4 °C. The supernatants were collected and centrifuged again under the same conditions. The protein concentration of the supernatants was measured by the method of Bradford [19].

### 2.3. Lipid Peroxidation

A lipid peroxidation kit from Calbiochem (437634, Calbiochem, EMD Biosciences Inc., San Diego, CA, USA) was used to measure the amount of malondialdehyde (MDA) and 4-hydroxy-2(E)-nonenal (4-HNE) as an index of the oxidative destruction of lipids. Data are presented as nmol (MDA+4-HNE) per mg protein.

### 2.4. Total Antioxidant Activity (TAA)

TAA was determined using the ABTS/H_2_0_2_/HRP method modified for tissue samples [20,21]. The results are expressed as equivalents of mg Trolox/mg protein.

### 2.5. Immunoblotting

The protein samples (100 μg) were prepared in Western blotting sample buffer (65.8 mM Tris-HCl, pH 6.8, 2.1% SDS, 26.3% (*w*/*v*) glycerol, 0.01% Bromophenol Blue). The 12% SDS-polyacrylamide gels were run and analyzed, as previously described [21,22,23]. Primary antibodies applied were—Beclin 1, perilipin (PLIN), p-mTOR, and mTOR from Santa Cruz Biotechnology (Santa Cruz, CA, USA), sequestosome-1 (SQSTM1/p62) from Cell Signaling Technology (Boston, MA, USA), Lysosomal acid lipase (LAL) antibody from Abcam (Cambridge, UK), and LC3 from MBL (Naka-ku Nagoya, Japan). Primary antibodies were diluted 1:1000 in blocking buffer. Goat anti-human β-actin antibody (Santa Cruz Biotechnology, Inc.) diluted at 1:1000 was always assayed as a loading reference. After washing in TBS-T (20 mM Tris-HCl, 150 mM NaCl, pH 7.4 and 0.05% Tween-20), the membranes were then incubated with the corresponding horseradish peroxidase-conjugated secondary antibody (Santa Cruz Biotechnology, Inc.) diluted at 1:2500. Binding of antibodies to their antigens was detected using the Western Blotting Luminol Reagent (sc-2048; Santa Cruz Biotechnology, Inc.), according to the manufacturer’s protocol. The results were calculated from at least three separate experiments for each antibody and were normalized to actin. Band intensity was quantified using the Quantity One 1D-analyzes software v. 5.5.1. (Bio-Rad Laboratories Inc., Hercules, CA, USA).

### 2.6. Statistical Analysis

Data are represented as mean ± standard error of mean (SEM). The Graphpad prism was used to perform ANOVA with a Tukey’s post-hoc test. * *p* < 0.05, ** *p* < 0.01, or *** *p* < 0.001 and # *p* < 0.05, ## *p* < 0.01, and ### *p* < 0.001 was considered statistically significant.

## 3. Results and Discussion

### 3.1. Melatonin Treatment Ameliorates the Lipid Effect Caused by a Fructose Diet

**Figure 1:**
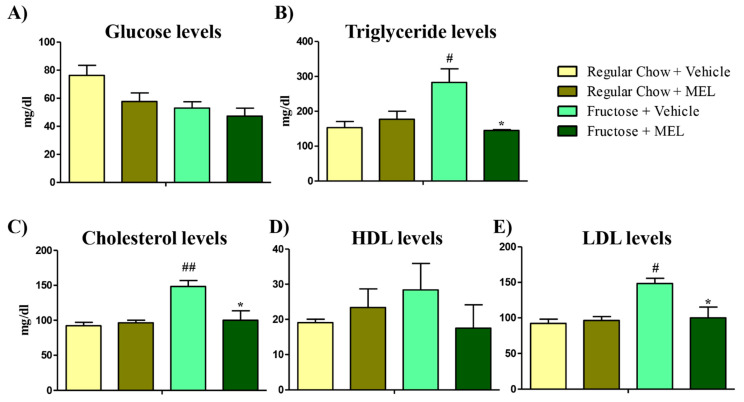
Fructose diet produces an imbalance in serum lipid markers that is ameliorated by melatonin. (**A**) Glucose levels in serum were measured in mg/dl from hamsters fed with regular chow or fructose with or without melatonin treatment (25 µg of melatonin for 15 days). (**B**) Triglycerides, (**C**) Cholesterol, (**D**) HDL, and (**E**) LDL levels in serum were measured in mg/dl from hamsters fed with regular chow or fructose with or without melatonin treatment. Bars are mean ± SEM. * *p* < 0.05 (differences caused by melatonin treatment) and # *p* < 0.05, ## *p* < 0.001 (differences caused by diet).

Syrian hamsters have a reproducible response to dietary manipulation [24]. Their lipid metabolism closely resembles that of humans, and unlike mice and rats, hamsters show cholesterol ester transport protein activity [25]. Fructose is an important nutritional factor in the development of the Metabolic Syndrome (MetS) in humans [2] and high fructose intake in hamsters also leads to the development of MetS-associated symptoms [18]. Syrian hamsters were fed with 60% fructose enriched food for 4 weeks, while control hamsters were fed with a regular chow diet. Then, half of the hamsters of each diet were injected with SQ melatonin for 15 days (25 µg of melatonin per hamster) [22]. Before sacrifice, blood was extracted, and biochemical determinations were performed. Glucose levels showed no significant differences between diets (Figure 1A). As hamster were not fasted before the blood extraction, changes were not expected either [3]. Fructose is a highly lipogenic sugar that is already associated with dyslipidemic symptoms induced by MetS [26]. Accordingly, triglycerides were increased with the fructose diet (Figure 1B, *p* < 0.05) and restored with the melatonin treatment (Figure 1B, *p* < 0.05). Total cholesterol was also increased (Figure 1C, *p* < 0.01), as well as LDL particles (Figure 1E, *p* < 0.05), whilst there was no significant compensation with the HDL cholesterol (Figure 1D). Melatonin restored total and LDL cholesterol levels (Figure 1C,E, *p* < 0.05). These results demonstrates that melatonin improves dyslipidemia in fructose-fed hamsters more efficiently than melatonin treatments in MetS’ patients [3]. This might be due to a higher dose in hamsters than in humans [3]. Melatonin’s role as a hypolipidemic is related to a decrease in intestinal cholesterol absorption [27] or inhibition of cholesterol biosynthesis and LDL-C accumulation [28] in rats. However, further studies need to be performed to decipher melatonin’s hypolipidemic role in hamsters and humans.

### 3.2. The Harderian Gland Is the Organ Most Affected by a Fructose Diet

**Figure 2:**
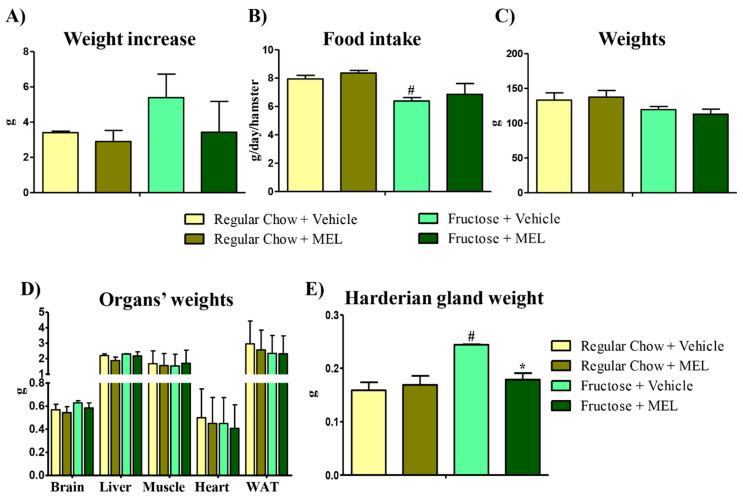
The Harderian gland is the organ most affected by a fructose diet. (**A**) Weight increase in grams of the hamsters fed with regular chow or fructose with or without melatonin treatment (25 µg of melatonin for 15 days). (**B**) Food intake measure in grams per day and hamster from all the above conditions. (**C**) Total weights in grams of all groups. (**D**) Different organs and (**E**) the Harderian gland weights in grams, from all the above conditions. Bars are mean ± SEM. * *p* < 0.05 (differences caused by melatonin treatment) and # *p* < 0.05 (differences caused by diet).

Weight and food intake was monitored during the experiment. Fructose-fed hamster weight increase tended to be higher (Figure 2A), but they were eating less (Figure 2B, *p* < 0.05), which underlined a metabolic unbalance [29]. At the end of the experiment, experimental groups did not show significant weight differences (Figure 2C). Reports showed that excessive fructose intake induced features of MetS in humans with and without obesity [6], which agreed with our results—fructose-fed hamster showed MetS symptoms like dyslipidemia, but not overweight. Then, we evaluated the different organ weights—brain, liver, muscle, heart, and white adipose tissue (WAT) showed no significative differences in any condition (Figure 2D). Nevertheless, the Harderian gland’s (HG) weight increased in the fructose-fed hamster (Figure 2E, *p* < 0.05) and this increase was suppressed with the melatonin treatment (Figure 2E, *p* < 0.05). HG cells accumulated fats in lipid droplets, which were active organelles in this organ [30]. Thus, HG weight increase could be due to an excessive lipid accumulation, impaired lipid metabolism, or defective lipophagy.

Dyslipidemia and increased lipid oxidation are symptoms of an early MetS [31]. The HG is a well-established model to study oxidative stress [22], and it showed the disease’s earliest hallmarks, with respect to other organs. Thus, HG is a good target to study both early stages of MetS and melatonin as a putative therapy. Our results indicated that this antioxidant rescued the weight changes and the dyslipidemia caused by fructose. We then decided to delve into the connections between MetS, oxidative stress, and defects in lipid metabolism and autophagy, using HG as a model. 

### 3.3. Melatonin Ameliorates Autophagic and Lipophagic Impairment in the Harderian Gland Caused by the Metabolic Syndrome.

**Figure 3:**
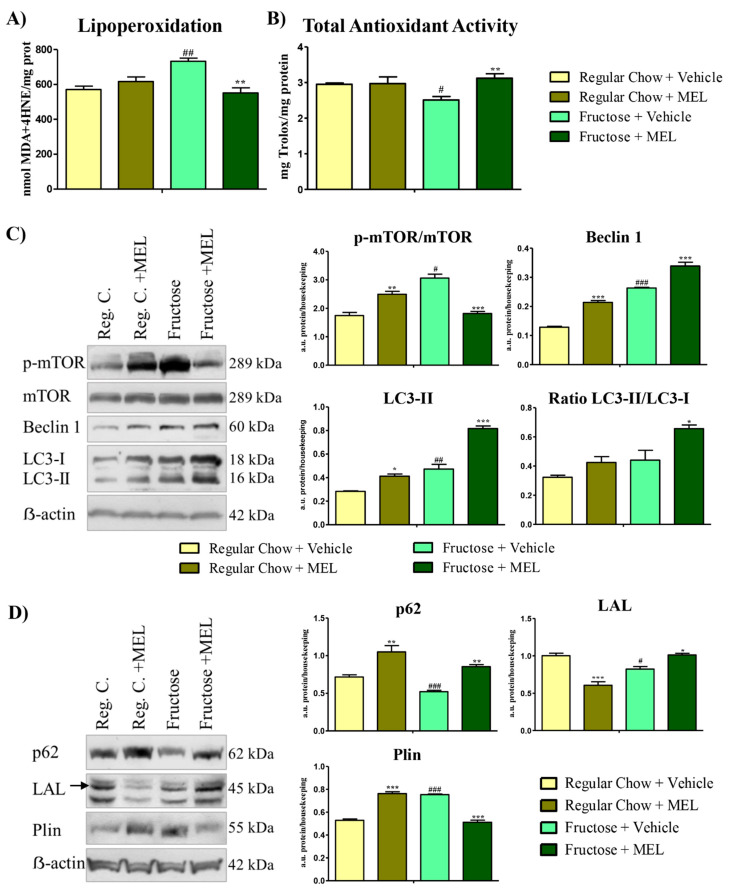
Melatonin ameliorates autophagic and lipophagic impairment in the Harderian gland in animals fed with fructose. (**A**) Harderian gland’s lipid peroxidation measured in nmols of 4-HNE+MDA/mg prot from hamsters fed with regular chow or fructose, with or without melatonin treatment (25 µg of melatonin for 15 days). (**B**) Harderian gland’s total antioxidant activity measured in mg Trolox/mg protein from hamsters from all the above conditions. (**C**) Autophagy pathway proteins and LC3-II/LC3-I ratio in Harderian gland’s homogenates from hamsters from all conditions (quantified in histograms of protein expression/actin expression and mTOR is represent as p-mTOR/mTOR). (**D**) Lipophagy-related proteins expression in Harderian gland’s homogenates from hamsters from all conditions (quantified in histograms of protein expression/actin expression). Bars are mean ± SEM. * *p* < 0.05, ** *p* < 0.01, *** *p* < 0.001 (differences caused by melatonin treatment) and # *p* < 0.05, ## *p* < 0.01, ## *p* < 0.001 (differences caused by diet).

Our previous results showed that HG was affected the most after fructose feeding. Early MetS is characterized by increased systemic markers of lipid oxidation [6]. Accordingly, the HG from fructose fed hamsters showed the highest levels of oxidated lipids (Figure 3A, *p* < 0.01). Melatonin treatment restored the lipoperoxidation levels (Figure 3A, *p* < 0.01). Prolonged state of oxidative stress results in reduction of antioxidative enzyme activities [3]. We also observed this effect in animals fed with fructose (Figure 3B, *p* < 0.05), a phenotype once again restored by a melatonin injection (Figure 3B, *p* < 0.01). Interestingly, melatonin had no effect in the regular chow-fed hamsters (Figure 3B).

Autophagy is key to eliminate damaged proteins, lipids, and organelles, favoring cellular survivability under oxidative stress environments or cellular damage in the HG [16,32], and in many other contexts [33,34]. We then wondered whether autophagy was also involved in this MetS scenario. HG from the fructose-fed hamster showed significant raised levels of the autophagy inhibitor mTOR (Figure 3C, *p* < 0.05), evaluated by the ratio of p-mTOR and mTOR expression (representative Westerns showed with their quantification). In a highly-nutrient state, mTOR is activated [35], which would fit with the fructose diet nutrient overload. Melatonin’s role in the mTOR pathway was not clarified and both activation and inhibition of the mTOR pathway were described in different contexts [36,37]. Melatonin treatment increased the mTOR activity in the regular chow-fed hamsters (Figure 3C, *p* < 0.01), which might be fostering the mTOR role in storing nutrients [38]. However, in the fructose-fed hamsters displaying an inflammatory environment caused by impaired lipid handling, melatonin treatment inhibited mTOR activity (Figure 3C, *p* < 0.001). This latter result would suggest that melatonin stimulates protective mechanisms when present in a toxic scenario [37]. We also found an increased expression in the autophagy inductor Beclin 1 produced by a fructose diet (Figure 3C, *p* < 0.001) and a further increase after melatonin treatment in both diets (Figure 3C, *p* < 0.001). LC3-II, a marker of autophagosomes, was also elevated (Figure 3C, *p* < 0.05). Likely, fructose diet induces an oxidative and inflammatory state that triggers autophagosome formation as cellular survival response, as previously described (15, 34). However, activated mTOR would keep autophagy inhibited [39]. LC3-II/LC3-I ratio was also measured to better understand the autophagic dynamics. We found that this ratio was increased in the fructose-fed glands when they were treated with melatonin (Figure 3C, *p* < 0.05). Conversely, and according to our experiments, melatonin treatment in the fructose-fed hamster triggers autophagosome formation (LC3-II and Beclin 1 are increased) and at the same time mTOR activity is reduced, which might allow a more efficient autophagy [32].

Autophagy is also involved in a plethora of other functions, such as an alternative energy source through a type of selective autophagy called lipophagy [40]. Moreover, fructose is a highly lipogenic sugar [26] that causes lipid metabolism impairment [25]. To decipher the role of this selective pathway, we analyzed the expression of proteins related with lipophagy in the Harderian gland. We previously described that p62, a key autophagic protein that mediates the selective specific degradation of protein aggregates and cytoplasmic bodies [41], is a key regulator of lipophagy in the HG [30]. Fructose diet reduced p62 expression (Figure 3D, *p* < 0.001), a phenotype partially recovered by melatonin treatment, which also increased p62 expression in a regular diet (Figure 3D, *p* < 0.01). Lipophagic processes were studied by assaying the expression of lysosomal acid lipase (LAL), which was reduced in the HG from fructose-fed hamsters (Figure 3D, *p* < 0.05) and was recovered by melatonin treatment (Figure 3D, *p* < 0.05). Finally, a specific lipid droplet marker, perilipin (Plin), was increased in fructose diet, implying more lipid droplets (Figure 3D, *p* < 0.001), which were reduced by melatonin treatment (Figure 3D, *p* < 0.001). Melatonin seemed to induce a different effect, depending on the diet in both LAL and Plin (Figure 3D, *p* < 0.001), which might depend on the availability of the autophagic machinery [40]. However, deep studies, i.e., through immunofluorescence and differential expression of more perilipins and its posttranslational modifications, would be required to understand how melatonin modifies lipophagy activity.

**Figure 4:**
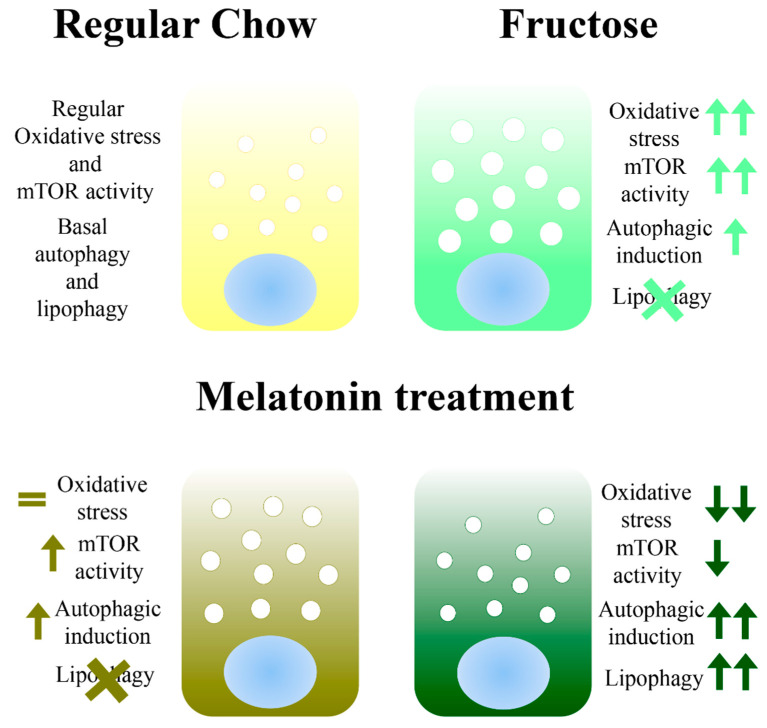
Proposed model—melatonin might have a dual role depending on the cellular situation. When cells are balanced, melatonin activates mTOR that promotes nutrient’s storage. When oxidative stress levels are high, melatonin activates autophagy and selective autophagy, through mTOR inhibition, which have a protective effect.

According to our results, damage produced in the HG by the fructose diet seem to induce autophagosome formation as an oxidative stress response. However, under nutrient-enrichment, mTOR is strongly activated and autophagy is consequently inhibited. Additionally, selective autophagy markers and lysosome lipase activity are diminished, and lipid droplets are accumulated. Melatonin might have a dual role depending on the cellular situation, when the cells are balanced, the melatonin activates the mTOR, which promotes the nutrient’s storage [38] as was observed by the Plin accumulation. However, when homeostasis is broken and oxidative stress levels are high, melatonin seems to activate autophagy and selective autophagy through mTOR inhibition [36], which would have a protective effect (Figure 4).

## 4. Conclusions

The Harderian gland is an ideal model to study early Metabolic Syndrome onset.

Melatonin improves dyslipidemia in a model of Metabolic Syndrome.

Fructose diet activates mTOR and inhibits autophagy in the Harderian glands.

Melatonin seems to activate lipophagy to ameliorate oxidative damage in an early Metabolic Syndrome model.

## Data Availability

The authors declare that the data supporting the findings of this study are available within the paper.
